# Supplementary material for: Personalizing the empiric treatment of gonorrhea using machine learning models
Source: PLOS Digit Health. 2024 Aug 14;3(8):e0000549. doi: 10.1371/journal.pdig.0000549 (PMC11324139; doi:10.1371/journal.pdig.0000549)
Supplement: S1 File — S1.1 GISP data and data pre-processing. S1.2 Hyperparameter tuning. S1.3 Bootstrap algorithm to calculate confidence intervals for performance measures. S1.4 Model specificity and sensitivity. S1.5 Calculating the effectiveness of personalized treatment recommendations and their impact on the unnecessary use of CRO/CFX. S2: Supplementary results. S2.1 Permutation importance and feature selection. S2.2 Model performance across target years. S2.3 Percentage receiving effective treatment and unnecessary CRO/CFX for logistic regression and random forest. S2.4 Leave-one-out cross-validation for logistic regression and random forest (DOCX) [file pdig.0000549.s001.docx]

**Supplementary Information**

# S1 Supplementary methods

## S1.1 GISP data and data pre-processing

Our dataset includes information about isolates, aggregated by surveillance site and sexual behavior, collected from 33 sentinel sites across five geographic regions in the US (Table [A).](#_bookmark4) To facilitate analysis, we disaggregated the data such that each observation represented an individual gonococcal isolate leaving no missing data with respect to the geographic location of the patient and the drug susceptibility. For the patient’s sexual orientation, the missing data came under the category of “Missing”, which also included the categories of “other or unknown”.

Table A: Clinics by region.

Region Clinics

Midwest Cleveland, Chicago, Kansas City, Cincinnati, St. Louis, Detroit, Minneapolis

Northeast New York City, Baltimore, Philadelphia

Southeast Greensboro, New Orleans, Miami, Birmingham, Fort Bragg, Atlanta, Richmond

Southwest Dallas, Albuquerque, Oklahoma City

West Portland, Phoenix, Honolulu, San Diego, San Francisco, Anchorage, Seattle, Denver, Las Vegas, Orange County, Los Angeles, Salt Lake City, Long Beach County

Applying one-hot encoding for categorical variables resulted in including thirteen features:

- 5 features for the location of the clinic where the sample was collected (Southwest, Southeast, West, Midwest, or Northeast);
- 4 features for whether the sample was taken from a man who has sex with men (“MSM”), a man who has sex with women (“MSW”), a man who has sex with men and women (“MSMW”), or whether the gender of the sexual partner was other, unknown, or missing (“Missing”);
- 1 feature for the prevalence of CIP resistance in the previous year across the entire region (“Regional CIP-R”),
- 1 feature for the change in CIP resistance in that region from the previous year (“Re- gional change in CIP-R”),
- 1 feature for the prevalence of CIP resistance in the patient’s clinic the previous year (“Local CIP-R”), and
- 1 feature for the change in prevalence of CIP resistance in that clinic from the previous year (“Local change in CIP-R”).
- 2 features for the number of observations the prevalence and trend data was calculated on (“Count >75” and “Trend > 75” respectively).

We used permutation importance to determine the set of features that maximizes auROC.

## S1.2 Hyperparameter tuning

To improve the performance of our models, we performed two rounds of hyperparameter tuning: one before and one after feature selection. The hyperparameters tuned for each model type are as follows:

- Logistic regression: solver, C (regularization strength), penalty
- Neural network: solver, activation function, alpha (regularization term), learning rate, hidden layer size
- Random forest: criterion, number of estimators, maximum depth of the tree, minimum samples before splitting, minimum samples per leaf

The ranges for the hyperparameters are shown in Table [B.](#_bookmark7)

Table B: Parameter Grid for Machine Learning Models

**Parameter Range**

Solver Newton c-g, lbfgs, liblinear Penalty l1, l2

C 0 - 100

Number of estimators 1 - 200

Max depth of tree 1 - 100

Min samples split 1 - 100

Min samples leaf 1 - 100

Solver lbfgs, sgd, adam Activation function tanh, relu

alpha 0-1

Learning rate constant, adaptive Hidden layer size 4-14

## S1.3 Bootstrap algorithm to calculate confidence intervals for performance measures

For each target year *t ∈ {*2005*,* 2006*,* *,* 2010*}*:

1. Train the ML model using the entire data collected during years [*t −* 6*, t −* 1] (sample size *N_t_*), including hyperparameter tuning and feature selection. Use the data collected during the target year *t* to calculate the estimate for the performance measure of

interest (e.g., auROC, and sensitivity and specificity curves). We use *O_t_* to denote this estimate.

1. For iterations *i* = 1 to 100:
   1. Sample *N_t_* observations with replacement from the data collected during years

[*t −* 6*, t −* 1] to create a bootstrapped dataset.

- 1. Use the same ML modeling, hyperparameter tuning, and feature selection tech- niques as used in Step 1 to train the ML model on the bootstrapped dataset.
  2. Calculate the *bootstrap* estimates for the performance measures of interest (e.g., auROC, and sensitivity and specificity curves) by applying the model to the data collected during the target year *t*. We use *B_t,i_* to denote this estimate.
  3. Calculate the estimated variation around *O_t_* (i.e., the difference between the *boot- strap* and *estimated* performances) as ∆*_t,i_* = *B_t,i_ − O_t_*.

1. Return [*O_t_ − δ_α/_*_2_*, O_t_ − δ*_1_*_−α/_*_2_] as the bootstrap confidence interval for the outcome of interest, where *δ_α/_*_2_ is the (1 *− α/*2)100th percentile and *δ*_1_*_−α/_*_2_ is the (*α/*2)100th percentile of *{*∆*_t,_*_1_*,* ∆*_t,_*_2_*, . . . ,* ∆*_t,N_ }*.

## S1.4 Model specificity and sensitivity

As additional measures of model performance, we calculated the model’s sensitivity (*α*) and specificity (*β*) for a given classification threshold (*p*) as:

**Sensitivity** = *α*(*p*) = true positives

true positives + false negative

**Specificity** = *β*(*p*) = true negatives

true negatives + false positives

(1)

(2)

Setting *p* = 0 is equivalent to categorizing all isolates as resistance to CIP, which is equivalent to 100% sensitivity and 0% specificity (i.e., correcting identifying 100% of resistant and 0% of susceptible isolates). Setting *p* = 1 is equivalent to categorizing all isolates as susceptible to CIP, which is equivalent to 0% sensitivity and 100% specificity (i.e., correcting identifying 0% of resistant and 100% of susceptible isolates).

## S1.5 Calculating the effectiveness of personalized treatment recommendations and their impact on the unnecessary use of CRO/CFX

When a predictive model is used to inform the first-line therapy for gonorrhea during target year *t*, the proportion of individuals with gonorrhea receiving effective treatment for a given classification threshold *p* is given by:

*θ*(*p*) = *α*(*p*)*µ_t_* + (1 *− µ_t_*) (3)

Table C: Proportion receiving effective treatment and unnecessary ceftriaxone or cefixime (CRO/CFX) under the three scenarios.

Scenario Proportion who receive ef- Proportion who receive

|  | fective treatment | CRO/CFX unnecessarily |
| --- | --- | --- |
| CIP-only | 1 - *µ_t_* | 0 |
| CRO/CFX only | 1 | 1 - *µ_t_* |
| Model | *θ*(*p*) | *ω*(*p*) |

and the proportion of individuals who unnecessarily receive CRO/CFX is given by:

*ω*(*p*) = (1 *− µ_t_*)(1 *− β*(*p*))*,* (4)

where *µ_t_* is the prevalence of CIP resistance for the target year *t*.

In the case where only CIP is prescribed, *ω*(*p*) = 0, individuals cannot be unnecessarily pre- scribed CRO/CFX if it is unavailable. This is equivalent to a classification threshold of *p* = 1, assuming all infections are CIP-susceptible. However, only patients with CIP-susceptible in- fections will receive effective treatment (Table [C).](#_bookmark10) Conversely, when only CRO/CFX is available, all patients will receive effective treatment (as there is negligible resistance to both antibiotics in the population). This is equivalent to a classification threshold of *p* = 0. However, all patients with CIP-susceptible infections do so unnecessarily (Table [C).](#_bookmark10) These two scenarios (“CIP” and “CRO/CFX”) represent the bounds of our model. When using predictions made by the ML models, intermediate values for both *ω*(*p*) and *θ*(*p*), depending on *p* and the prevalence of CIP resistance in the training dataset, as described in the above equations.

To estimate treatment effectiveness (*θ^′^*) and the unnecessary use of CRO/CFX (*ω^′^*) under

*standardized guidelines*, we used the following approach. During the period 2005–2007, the

guidelines recommended treatment with CIP, CRO, or CFX. In an extreme scenario where all patients were prescribed CIP, 100(1 *− µ_t_*)% of patients would have received effective treatment and none would have been treated unnecessarily with CRO/CFX. This scenario is

represented by point B in Fig A[.](#_bookmark0) In an alternative extreme scenario, where all patients were prescribed CRO/CFX, all patients would have been prescribed effective treatment but

100(1 *− µ_t_*)% of patients would have been unnecessarily treated with CRO/CFX. This is

represented by point A in Fig [A.](#_bookmark0)

The dashed line that connects points A and B represents the effectiveness of standardized guidelines and their impact on the unnecessary use of CRO/CFX if an intermediate portion of patients were prescribed CIP and the rest were prescribed CRP/CFX. The formula of the dashed line is given by

*ω′* = 1 *− µ_t_* *µ*

*t*

*µ_t_*

+ *θ^′^ −* 1 (5)

In 2005 and 2006, when the CDC changed its recommendations for California, Hawai‘i, and MSM, the value of *ω* is calculated as a weighted average of the calculated values from

observations falling within those specific criteria (proportion *ρ*):

*ω* = *ρω_ρ_* + (1 *− ρ*)*ω_ρ_′* (6)

The vertical distance between the dashed line and the solid curve represents the reduction in the unnecessary use of CRO/CFX by following the recommendations of the predictive model for a given treatment effectiveness threshold. For example, the distance between points C and D measures the reduction in the unnecessary use of CRO/CFX when both the standardized guideline and the predictive model result in 98% of individuals with gonorrhea receiving effective therapy.


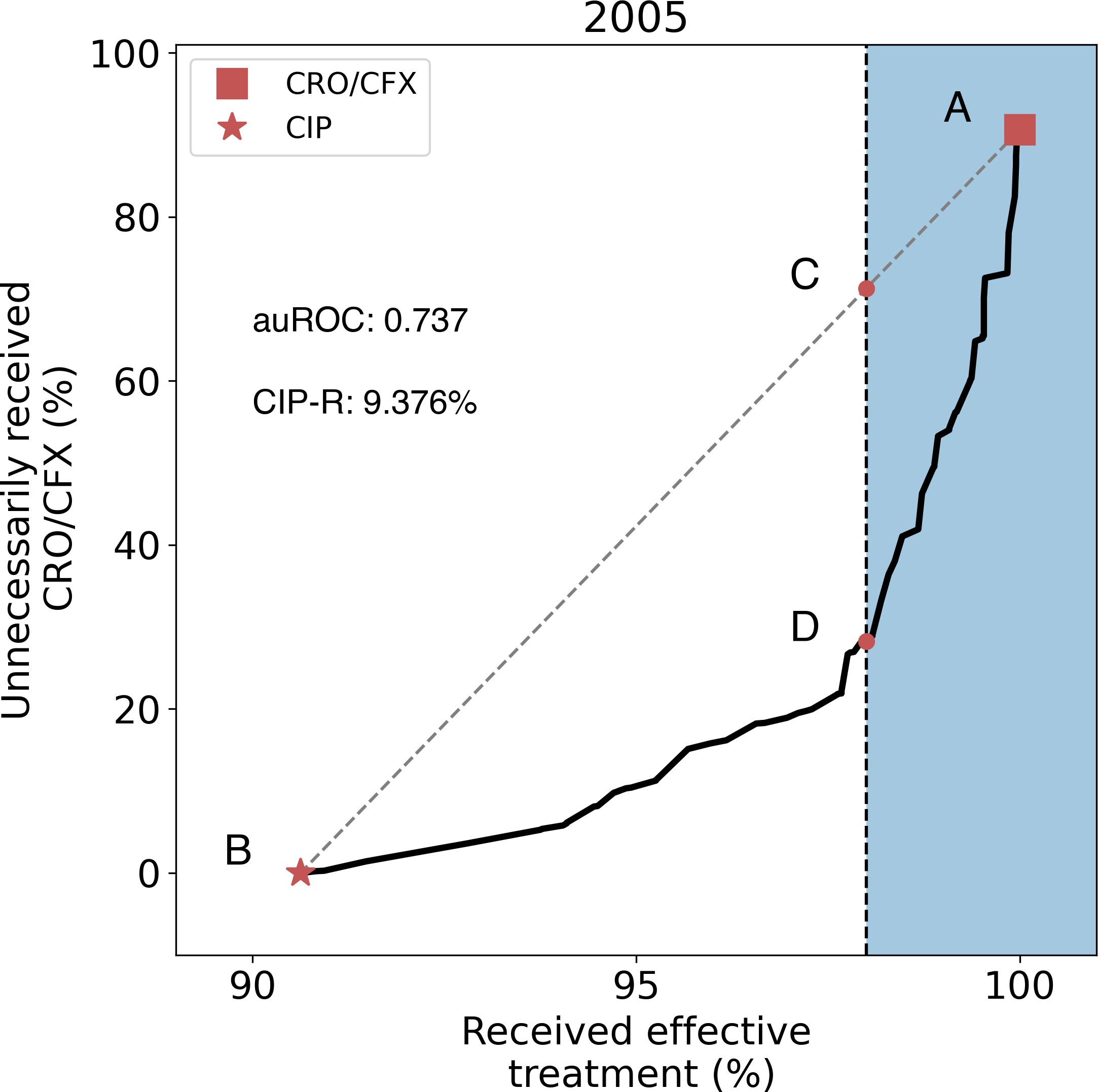


Fig A: **Calculation of the reduction in unnecessary use of ceftriaxone (CRO) and cefixime (CFX).**

# S2 Supplementary results

## S2.1 Permutation importance and feature selection

As part of the model development procedure, we conducted permutation importance to identify features that improved the model’s auROC. Only features that did not decrease

the training auROC were used in the final models. In the random forest models, the most important features across all years included whether the patient was MSM or MSW, the local prevalence of CROP-R, and the change in that local prevalence. However, across all years, the majority of features were included.

For the logistic regression model, the most important features were whether the patient was MSW or whether the data on sexual partners was “Other/Missing”, whether the sample was located from a clinic located in the West and the local change in CIP-R prevalence.


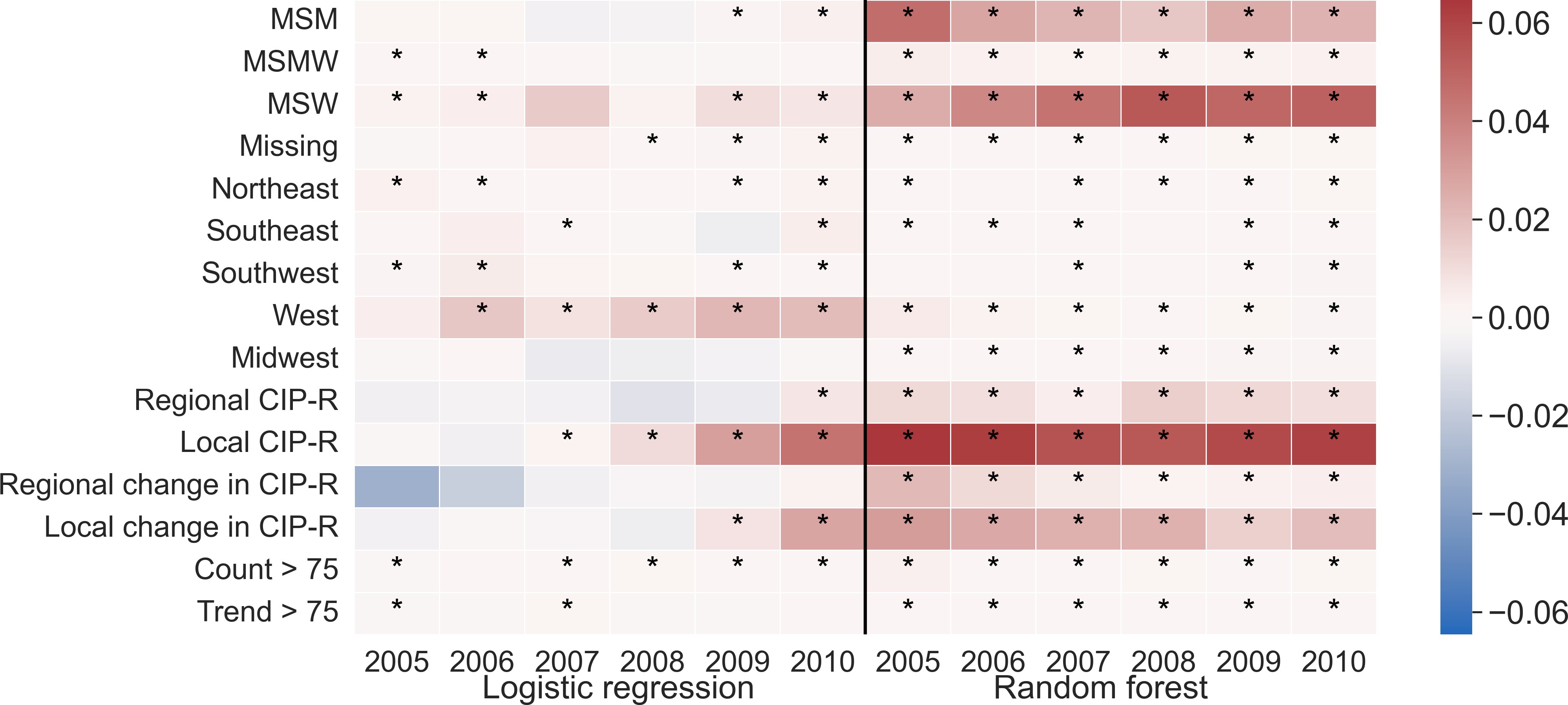


Fig A: **The contribution of features to the auROC score across each model and target year.** The color bar represents the contribution of each factor, measured by the feature importance score provided by the permutation importance algorithm. Only features with a positive effect on the training auROC were used in the final models. These features are represented by ’*’. CIP-R is the prevalence of resistance to ciprofloxacin.

## S2.2 Model performance across target years

We also evaluated the performance of predictive models developed here based on their F1 score and Matthews correlation coefficient (MCC)1, which are displayed in Fig [B).](#_bookmark2) The adjusted MCC is a correlation coefficient between the models’ prediction and the target; a value of 0 means no correlation, while a value of 1 indicates perfect correlation. A high F1 score indicates the model has high precision (the number of true positives divided by the total number of positives) and recall (the number of true positives divided by the num- ber of positives predicted by the model). The neural network and random forest models demonstrated comparable performance across all target years.


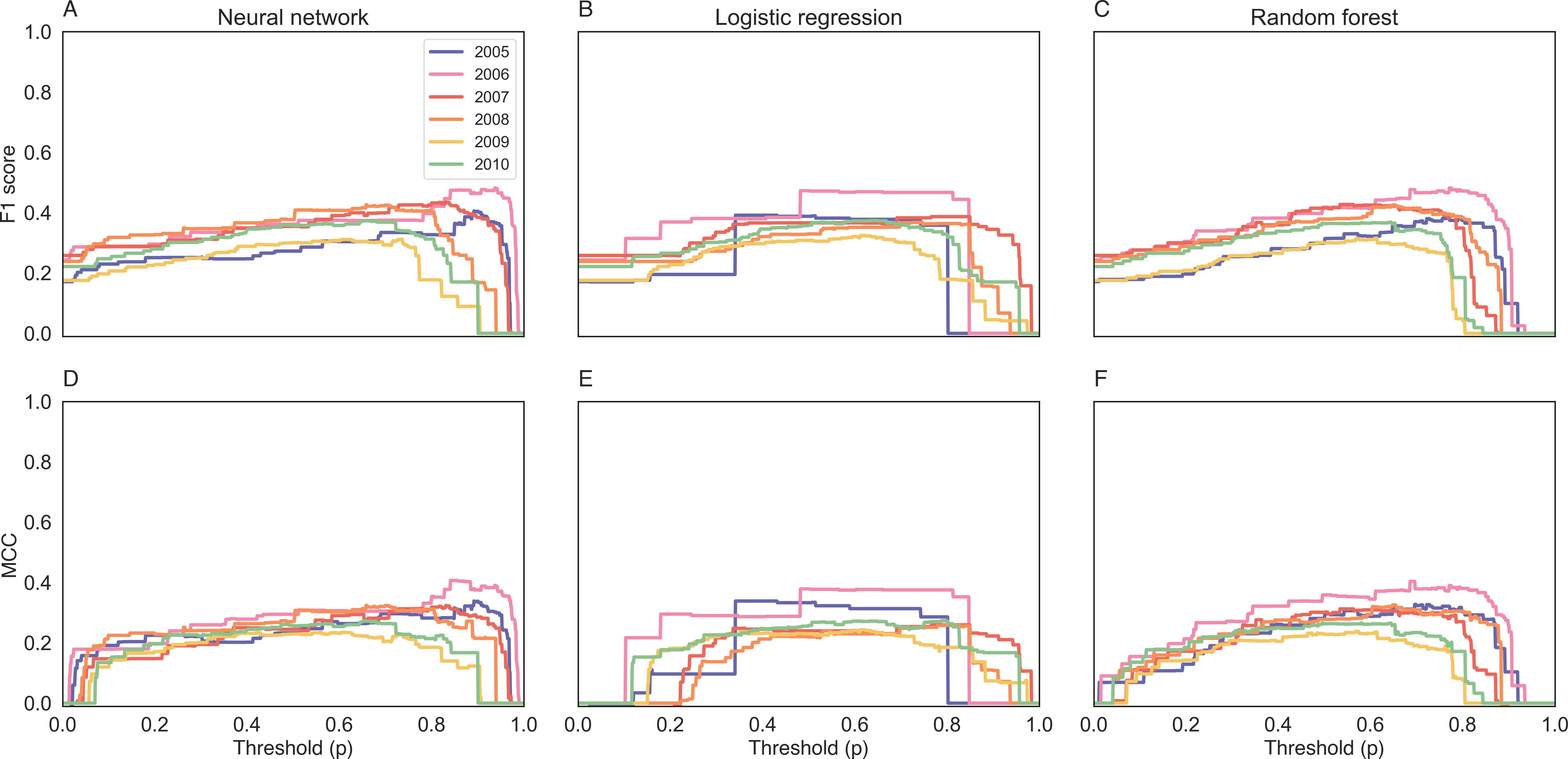


Fig B: **Performance metrics (F1 score and Matthews Correlation Coefficient, MCC) across models and target years.**

**S2.2.1 Sensitivity and specificity**

The classification threshold (above which we determined that patients had a CIP-non- susceptible infection) has a strong influence on both the specificity and the sensitivity of each of our ML models (Figs C-E) At lower classification thresholds, the model has high sensitivity but low specificity, meaning that the model can correctly classify patients as having a CIP-non-susceptible infection, but cannot detect those with a CIP-susceptible infec- tion. Conversely, as the classification threshold increases, the models get better at predicting CIP-susceptible infection, but cannot predict CIP-non-susceptible infections as accurately.

Across all models, therefore, there is a trade-off between sensitivity and specificity: as a model is better at detecting CIP-non-susceptible infections, it gets worse at detecting CIP- susceptible ones.


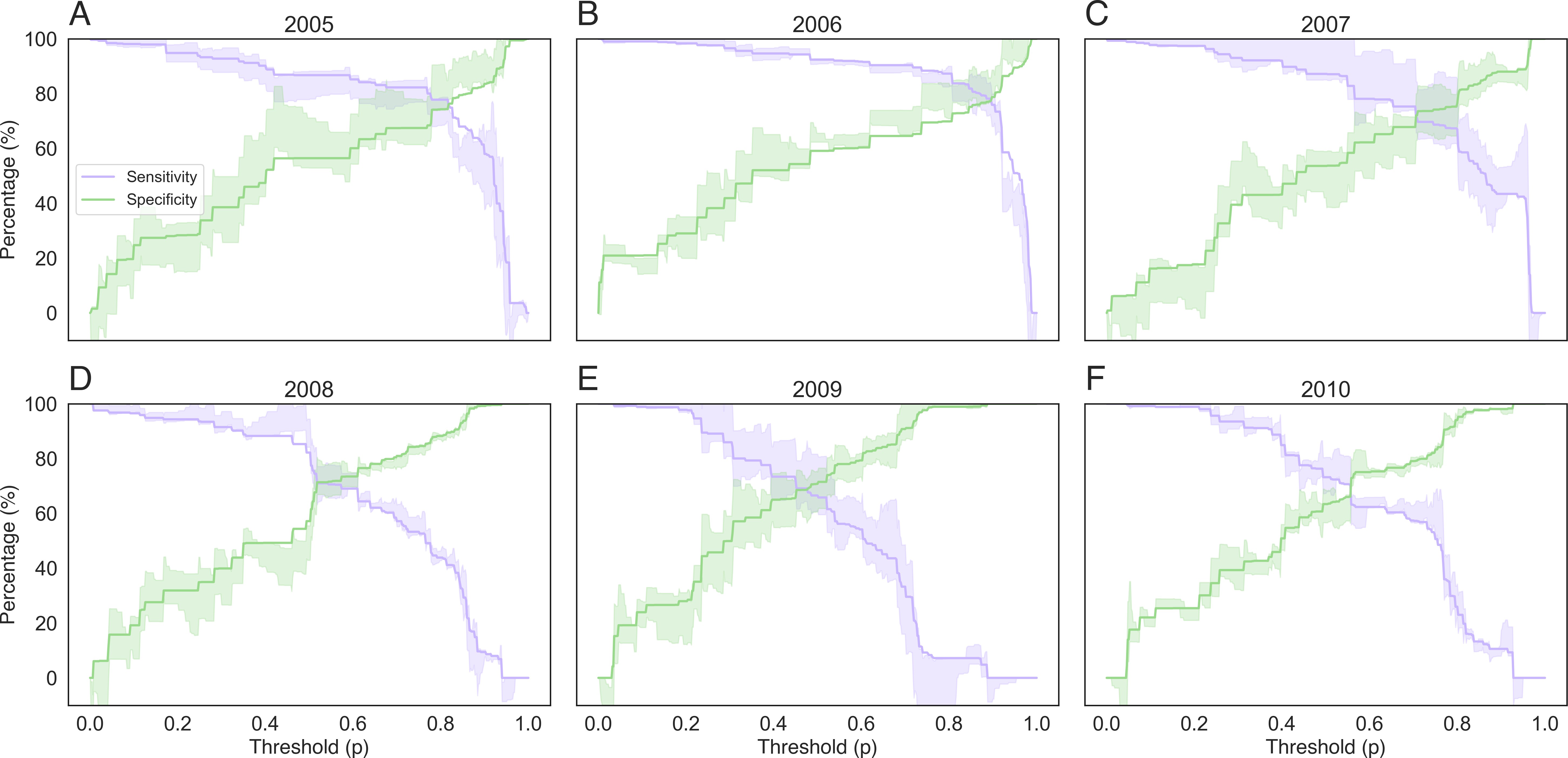


Fig C: **Sensitivity and specificity of the neural network model for 2005–2010.**


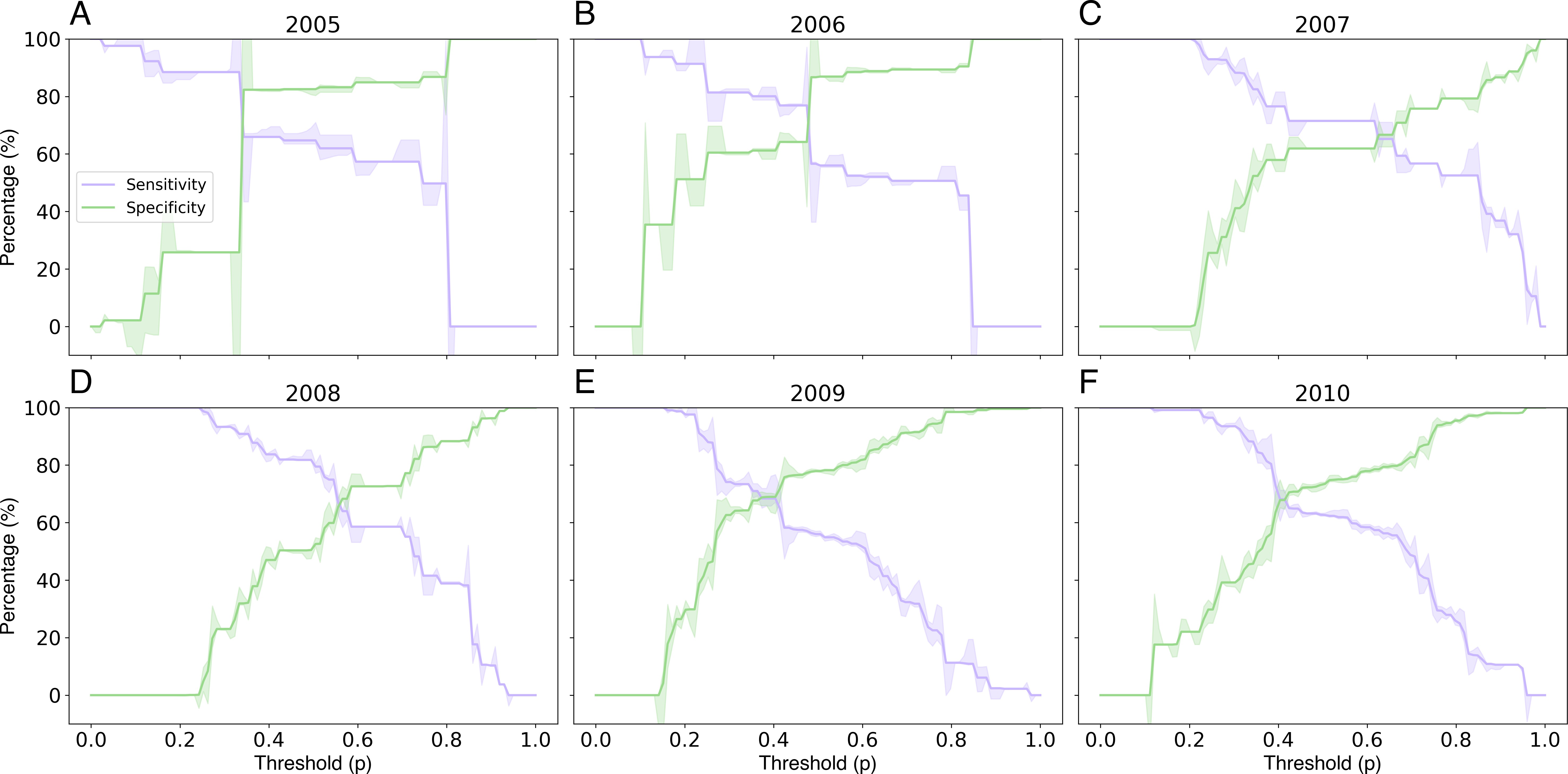


Fig D: **Sensitivity and specificity of the logistic regression model for 2005– 2010.**


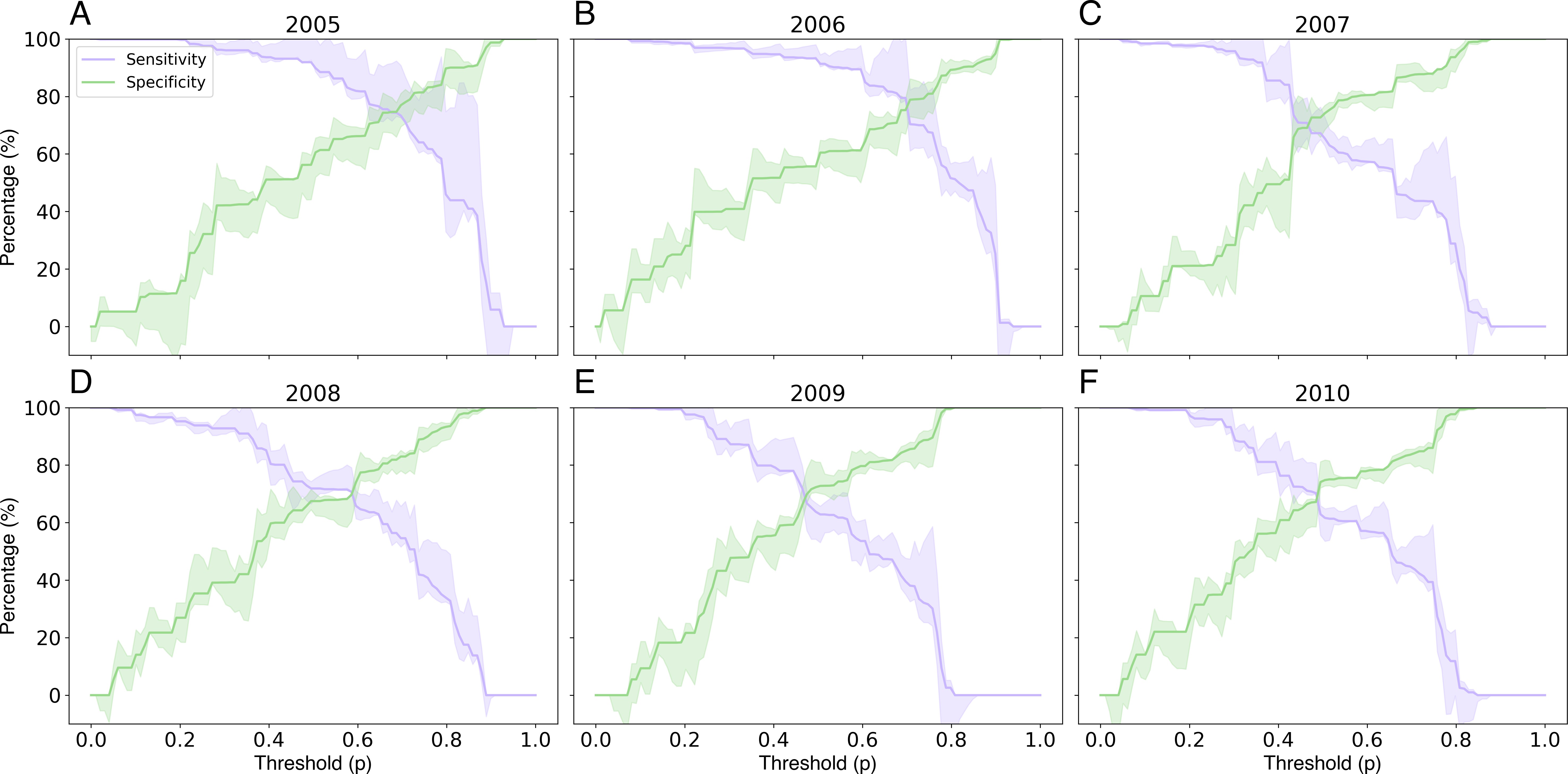


Fig E: **Sensitivity and specificity of** **the random forest model for 2005–2010..**

## S2.3 Percentage receiving effective treatment and unnecessary CRO/CFX for logistic regression and random forest

Across both the random forest and logistic regression models (Figs [F-G),](#_bookmark14) the predictive model reduced the number of patients who were unnecessarily prescribed CRO/CFX. This came with a decrease in the number who received effective treatment, though this was comparatively small.


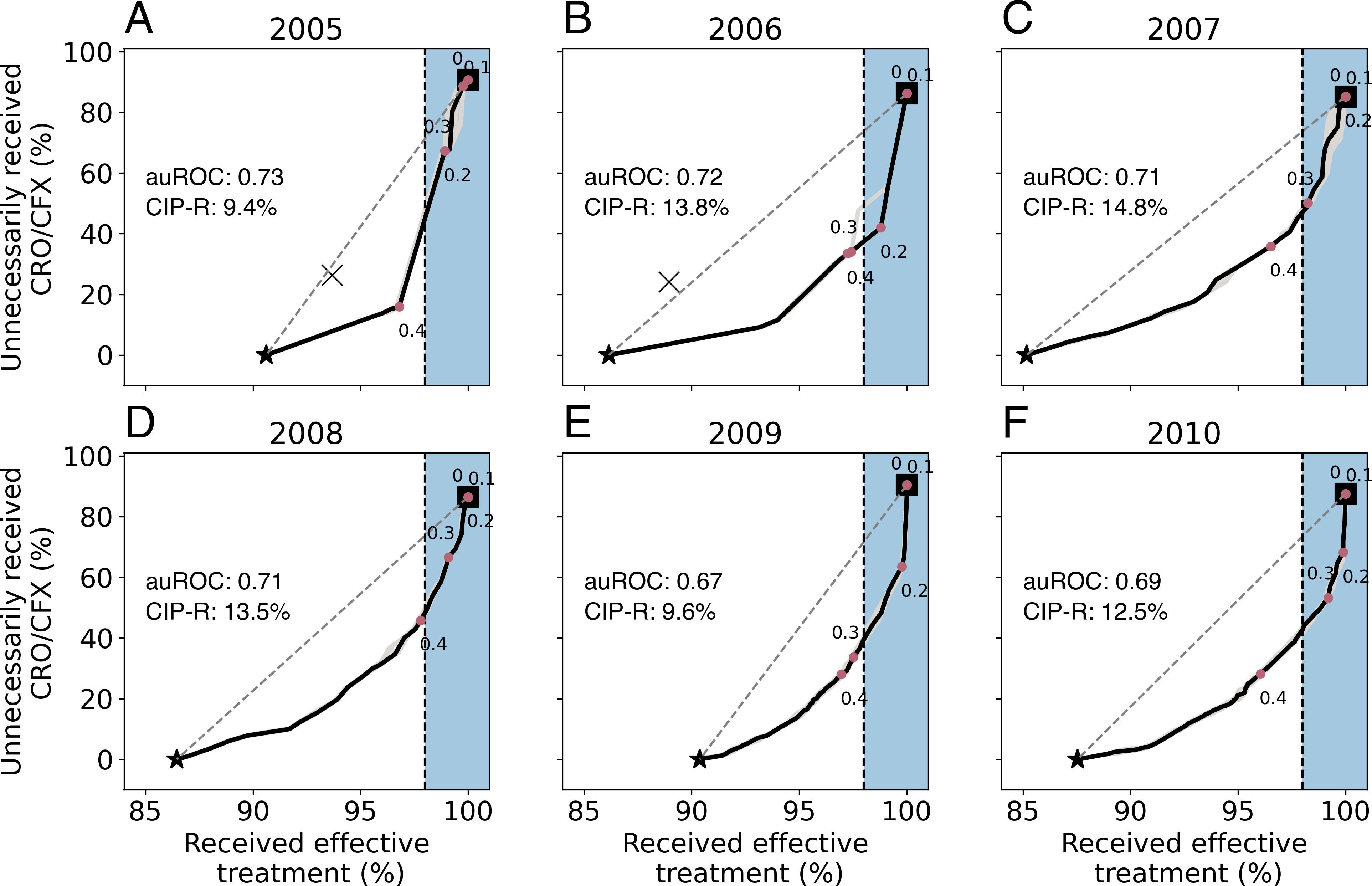


Fig F: **The proportion of patients** **with gonorrhea receiving effective treat- ment or being unnecessarily treated with CRO/CFX when CIP could have been effective. The personalized treatments are informed by logistic regression mod- els.** See the caption of Fig [3](#_bookmark2) in the main text for additional details.


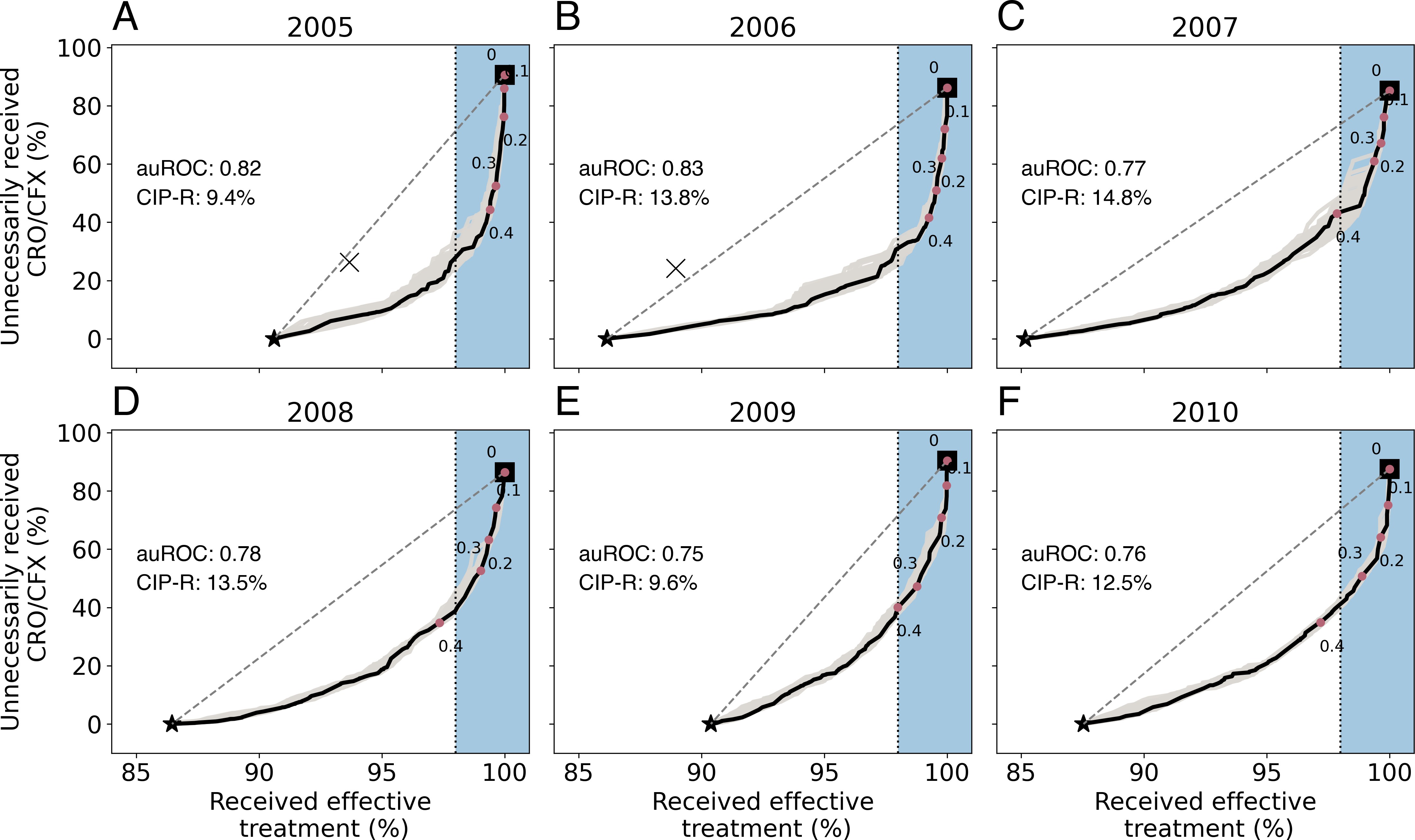


Fig G: **The proportion of patients** **with gonorrhea receiving effective treat- ment or being unnecessarily treated with CRO/CFX when CIP could have been effective. The personalized treatments are informed by random forest models.** See the caption of Fig [3](#_bookmark2) in the main text for additional details.

## S2.4 Leave-one-out cross-validation for logistic regression and random forest

As with the neural network model, for both the logistic regression model and the random forest, the aggregate model has good generalizability to each clinic (Figs [H-I).](#_bookmark16)


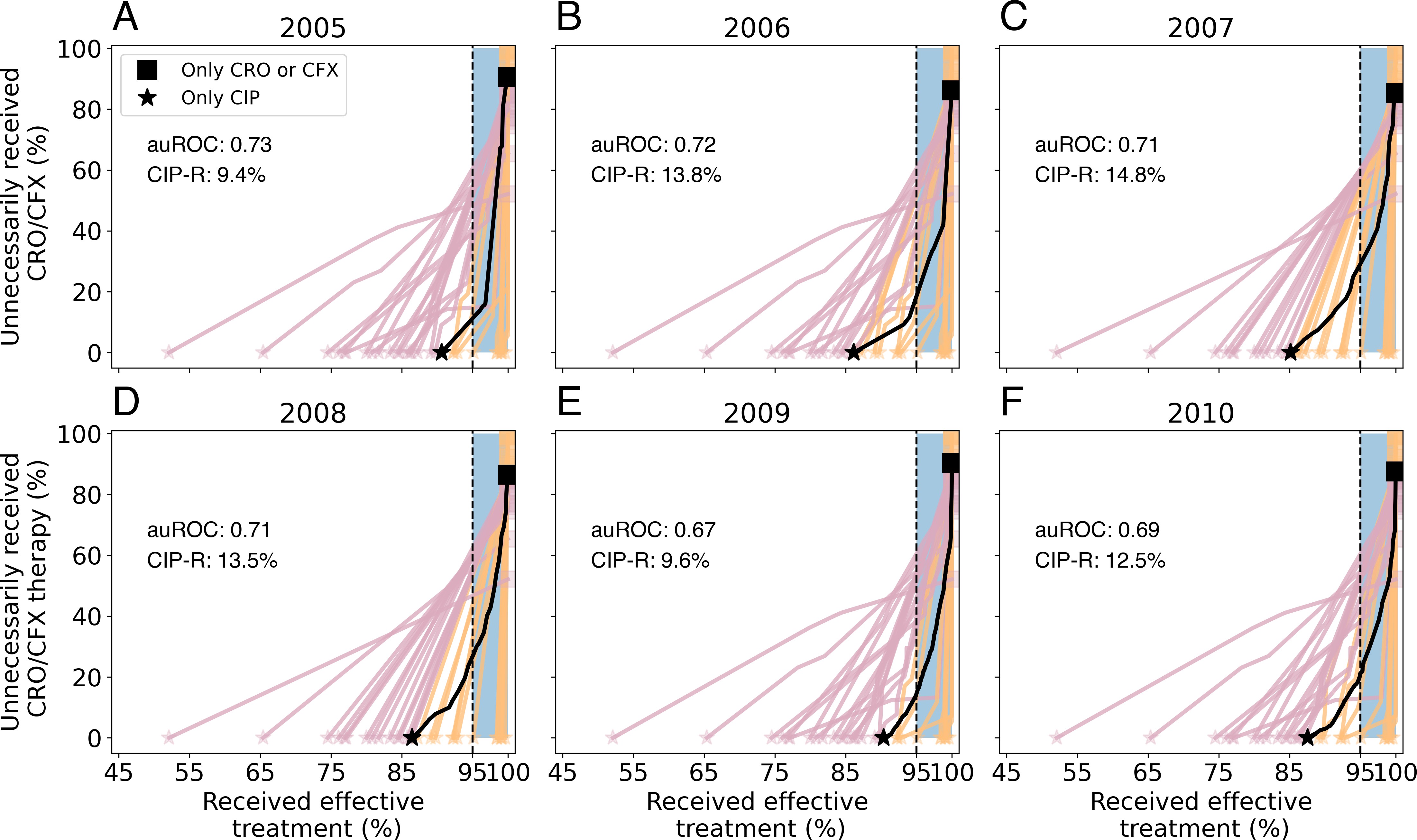


Fig H: **Leave-One-Out Cross Validati****on (LOOCV) for logistic regression mod- els.** See the caption of Fig [4](#_bookmark3) in the main text for additional details.


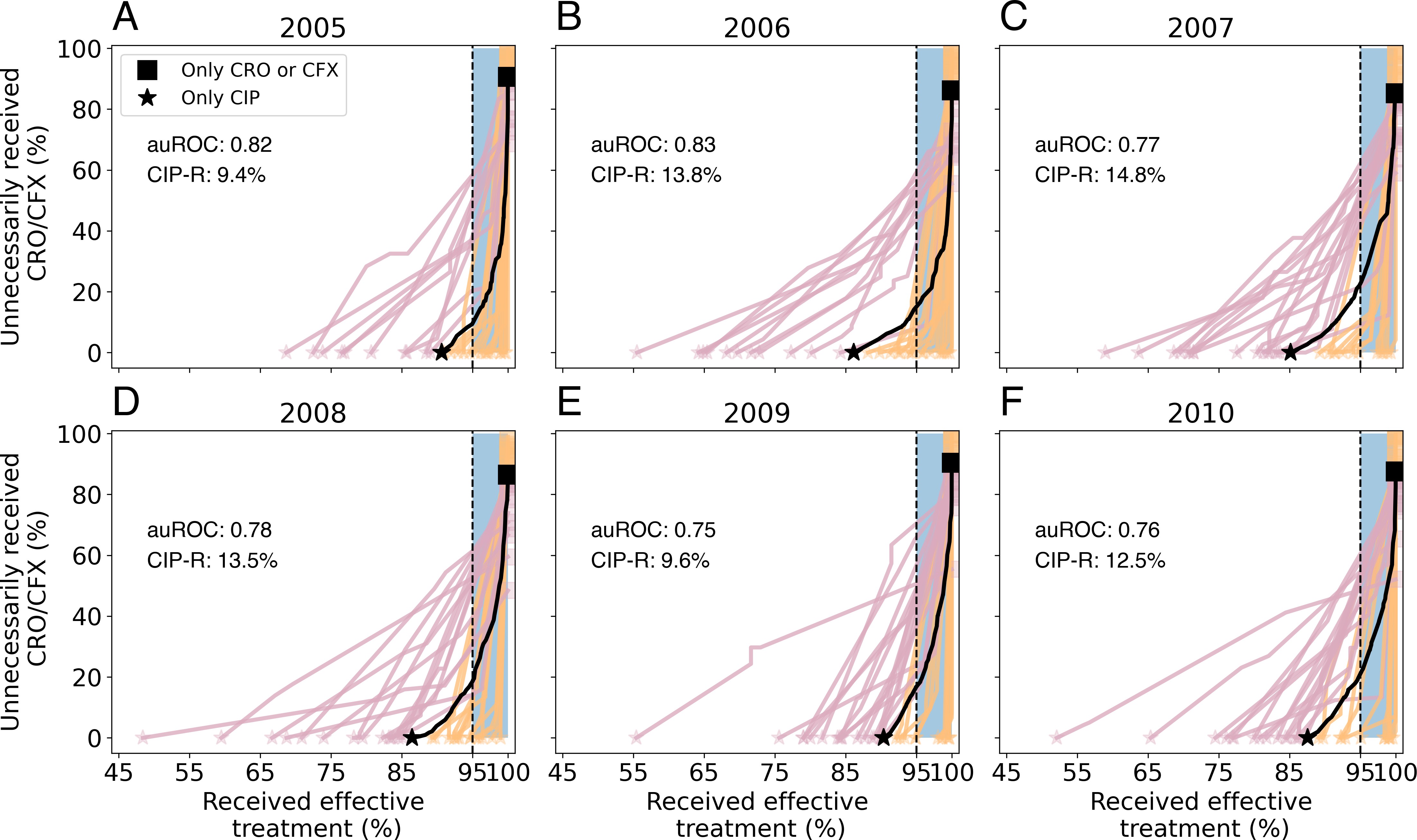


Fig I: **Leave-One-Out Cross V****alidation (LOOCV) for random forest models.**

See the caption of Fig [4](#_bookmark3) in the main text for additional details.

# References

1 Davide Chicco and Giuseppe Jurman. The advantages of the matthews correlation coeffi- cient (mcc) over f1 score and accuracy in binary classification evaluation. *BMC genomics*, 21:1–13, 2020.
