# Supplementary material for: Personalizing the empiric treatment of gonorrhea using machine learning models
Source: PLOS Digit Health. 2024 Aug 14;3(8):e0000549. doi: 10.1371/journal.pdig.0000549 (PMC11324139; doi:10.1371/journal.pdig.0000549)
Supplement: Attachment — Submitted filename: Response_to_Reviewers.docx [file pdig.0000549.s003.docx]

**Rachel Murray-Watson, Ph.D.**

Postdoctoral Associate

Yale School of Public Health

*courier*

350 George Street, Room 308

New Haven, CT 06511

rachel.murray-watson@yale.edu

May 29, 2024

Dear Professor Al-Jumeily:

Thank you for the opportunity to submit a revised version of our manuscript (tracking number: PDIG-D-24-00036): “Personalizing the empiric treatment of gonorrhea using machine learning models.”

We would like to thank reviewers for their constructive comments, which have contributed substantially to improving the content and the presentation of our manuscript. We have addressed each of the suggestions and concerns, and we have provided point-by-point responses in the attached response letter.

Thank you again for your consideration of this revised manuscript. We look forward to your evaluation and are available if questions arise.

Sincerely,

Rachel Murray-Watson, on behalf of co-authors.

# Responses to Reviewer 1

**Abstract**

1. **A clear overview of study objectives, methods, results, and conclusions.**
2. **The significance of personalized treatment approaches is effectively highlighted.**
3. **Specific data points were provided, but numerical values for improvements with personalized treatments compared to guidelines would enhance clarity.**

**Introduction**

1. **Thorough contextualization of the research problem within the broader issue of AMR gonorrhea.**
2. **A comprehensive overview of empirical treatment practices and the limitations of standardized guidelines.**
3. **The historical context of treatment recommendations adds depth, but mentioning the limitations of current practices could improve discussion.**

Response: Thank you for this feedback. We have added the following to the introduction:

“To ensure the effectiveness of empiric first-line therapy, guidelines recommend antibiotics with a low national prevalence of resistance (usually <5%) for all patients with gonorrhea.^6–8^ However, reports of substantial variation in the AMR prevalence and trend among different geographic regions or population subgroups^9–13^. Given the empiric nature of treatment, these variations led to large disparities in the number of patients receiving effective treatment. Such concerns resulted in revised guidelines that accounted for these variations.”

**Methods**

1. **Detailed and well-organized explanation of the dataset, study settings, predictors, outcomes, and model development.**
2. **Justification for feature selection was provided, but the inclusion of handling missing data and potential biases would be beneficial.**

Response: We thank the reviewer for this comment. With respect to the geographic location of the patient and the drug susceptibility, there was no missing data as our individual-level derived from data aggregated at the level of the test clinic. For the patient’s sexual orientation, the missing data came under the category of “Missing”, which also included the categories of “other or unknown”. To make this clear to the readers, we added the following to Methods:

“Two variables (region and sexual orientation) were nominal categorical variables, and we used one-hot encoding to include them in our analysis (§S1.1 of SI). The geographic location was available for all individuals. For sexual orientation, we coded entries with missing, other, or unknown as "missing" and kept them in the analysis. We standardized all continuous variables (the previous year’s regional and clinical prevalence of CIP resistance and the change in regional and clinical prevalence).”

We have added the following to the SI:

“To facilitate analysis, we disaggregated the data such that each observation represented an individual gonococcal isolate leaving no missing data with respect to the geographic location of the patient and the drug susceptibility. For the patient’s sexual orientation, the missing data came under the category of “Missing”, which also included the categories of “other or unknown”.”

And to acknowledge potential biases, we added the following as a limitation to the Discussion section:

“Fourth, between 2000–2010, GISP included isolates only from the first 25 men at each surveillance site presenting with urethral gonorrhea. However, considering the potential variance in resistance dissemination among population groups, the performance of our ML models developed in this study may differ for other sexual behavior populations or individuals infected at anatomical sites beyond the urethra.”

3. **A brief rationale for choosing the neural network model as the primary focus would enhance clarity.**

Response: The NN provided the most consistent performance for each target year of interest across all metrics that we considered. Temporal consistency was important when choosing our models as it means that the models can respond to changes in trends of CIP-R resistance. We have included the following sentence in the results:

“Based on these metrics, the neural network model had the highest and most consistent performance scores across target years, indicating that it could respond to changes in the prevalence of CIP-R across time. We, therefore, focus the remainder of our analyses on neural network models, though results for the other ML models are shown in S2 of SI.”

**Results**

1. **Effective presentation of key findings, including the prevalence of CIP resistance, model performance metrics, and impact of personalized treatment.**
2. **Used figures and tables enhanced readability, and more detailed explanations of model performance metrics would aid interpretation.**

Response: Thank you for your suggestion. We have added the following to the methods section:

“Models developed for each target year had an auROC greater than 0·5, indicating that they were better than random chance at predicting reduced susceptibility to CIP (Figure 1). The higher the auROC values, the better the model was at distinguishing between ciprofloxacin-susceptible and resistant cases.”

And the following to the SI under section “S2.2 Model performance across target years”:

“The adjusted MCC is a correlation coefficient between the models’ prediction and the target; a value of 0 means no correlation, while a value of 1 indicates perfect correlation. A high F1 score indicates the model has high precision (the number of true positives divided by the total number of positives) and recall (the number of true positives divided by the number of positives predicted by the model).”

# Responses to Reviewer 2

1. **The abstract should be rewritten to show the findings of the study – the present abstract only communicates background and procedural information with minimal results.**

Response: Thank you for this feedback. We have restructured the Abstract, and in particular we added the following to the Results section:

“Compared with standardized guidelines, the personalized treatments could have replaced 33% of CRO and CFX use with CIP while ensuring that 98% of patients were prescribed effective treatment during 2005–2010. The models maintained their performance over time and across geographic regions.”

2. **Some characteristics such as age group and ethnicity are important to include. Why have they not been considered.**

Response: Thank you for addressing this lack of data. These variables were not included as they are not made available as part of the GISP dataset. To clarify this in the paper, we have added the following to the “Predictors and outcomes” section of the methods:

“These features were based on data made available as part of the GISP survey. Other variables of interest, such as age, or ethnicity, were not available as part of this dataset.”

We have also discussed this as a limitation in Discussion:

“Third, we included a limited number of features in our model (namely, the sexual behavior of the patient, geographic region, the prevalence of CIP resistance, and the change in regional prevalence of CIP resistance). However, other socio-demographic and health factors are believed to be associated with having a drug-resistant gonorrhea infection.13, 19, 22–28 Including these variables may enhance our model’s predictive power and could be considered in future analyses. Nevertheless, despite the limited range of features, our ML models displayed reasonable predictive capabilities and demonstrated the potential to reduce the unnecessary use of CRO/CFX. Moreover, the selected features can be easily obtained at the point of care, facilitating their implementation in clinical settings.”

3. **Add list of definition to the methods**

Response: Thank you for your feedback. We have included the following definitions to a new section, “Abbreviations” but also happy to include there elsewhere per recommendation from the Editors:

- GISP: Gonococcal Isolate Surveillance Project
- CIP: ciprofloxacin
- CRO: ceftriaxone
- CFX: cefixime
- TRIPOD: Transparent Reporting of a multivariable prediction model for Individual Prognosis Or Diagnosis
- auROC: area under the Receiver Operating Characteristic curve
- MCC: Matthews' Correlation Coefficient
- LOOCV: Leave One Out Cross Validation

4. **What type of neural networks were used?**

Response: Thank you for highlighting this oversight. The neural networks were multilayer perceptrons (MLPs). We have added the following to the Model development section of the methods:

“For each year, we considered three supervised Machine Learning (ML) models: a multilayer perceptron (neural network), a logistic regression…”

5. **Page 5, there should not be a come after Python.**

Response: Thank you for spotting this error. It has now been corrected.

6. **Justify the use of 10 fold cross validation**

Response: We chose 10-fold cross validation, as these are empirical data to show that its use leads to models that do not have high bias or variance. Thus, it became a standard in the field. We have now added the following justification and reference to the “Model development” section of the methods:

“ten-fold cross-validation, which has been shown to reduce the bias and variance of predictive models ^17^”

7. **Page 7, on what basis was the target value chosen?**

Response: We chose the value of 98% as this was an improvement over the current CDC target value of 95% (i.e. only changing the front-line antibiotic treatment once 5% of infections are resistant to that treatment.

8. **One of the limitations that should be highlighted is explainabilty of the model**

Response: Thank you for mentioning this limitation of machine learning models. In the third limitation we discuss, we have added the following text:

“However, we do acknowledge that knowing the features which are important to the models’ prediction does not indicate how those features are being used by the model. The explainability of machine learning models is an area of ongoing and much-needed research to facilitate the use of machine learning models in practice.^29,30^”
