# Supplementary material for: Personalizing the empiric treatment of gonorrhea using machine learning models
Source: PLOS Digit Health. 2024 Aug 14;3(8):e0000549. doi: 10.1371/journal.pdig.0000549 (PMC11324139; doi:10.1371/journal.pdig.0000549)
Supplement: Attachment — Submitted filename: Reviewer comments.docx [file pdig.0000549.s002.docx]

**Manuscript Number: PDIG-D-24-00036**

**Title: Personalizing the empiric treatment of gonorrhea using machine learning models**

**Abstract**

- A clear overview of study objectives, methods, results, and conclusions.
- The significance of personalized treatment approaches is effectively highlighted.
- Specific data points were provided, but numerical values for improvements with personalized treatments compared to guidelines would enhance clarity.

**Introduction**

- Thorough contextualization of the research problem within the broader issue of AMR gonorrhea.
- A comprehensive overview of empirical treatment practices and the limitations of standardized guidelines.
- The historical context of treatment recommendations adds depth, but mentioning the limitations of current practices could improve discussion.

**Methods**

- Detailed and well-organized explanation of the dataset, study settings, predictors, outcomes, and model development.
- Justification for feature selection was provided, but the inclusion of handling missing data and potential biases would be beneficial.
- A brief rationale for choosing the neural network model as the primary focus would enhance clarity.

**Results**

- Effective presentation of key findings, including the prevalence of CIP resistance, model performance metrics, and impact of personalized treatment.
- Used figures and tables enhanced readability, and more detailed explanations of model performance metrics would aid interpretation.

**Discussion**

- Accepted with minor revisions.
- Comprehensive interpretation of results, contextualized within the broader literature on AMR gonorrhea and empirical treatment practices.
- Limitations are appropriately acknowledged, but further discussion on implications for clinical practice and policy integration would be beneficial.
- Well-conducted study addressing important public health issues.
- Findings contribute valuable insights into the potential of personalized treatment approaches.
